# Surgical treatment of an invasive thymoma extending into the superior vena cava and right atrium

**DOI:** 10.1186/1477-7819-12-6

**Published:** 2014-01-08

**Authors:** Yong-Qiang Dong, Jiang-Shui Liang, Xiao-Ming Zhang, Shui-Bo Zhu, Jia-Hang Xu, Tao Ji, Gui-Lin Yin

**Affiliations:** 1 Department of Cardio-Thoracic Surgery, Wuhan General Hospital of Guangzhou Military Command, Wuhan, Hubei 430070, China; 2 Department of Cardio-Thoracic Surgery, Southern Medical University Affiliated Clinical College of Wuhan, Wuhan, Hubei 430070, China

**Keywords:** Invasive thymoma, Superior vena cava, Right atrium, Autologous pericardial ‘Y’ conduit

## Abstract

Although invasive thymoma commonly infiltrates neighbouring mediastinal structures, its extension into the superior vena cava (SVC) and consequent SVC occlusion are rare. In such cases, the urgent removal of the thymoma and radical resection of the infiltrated SVC representreasonable options, since induction therapy is time-consuming and useless for symptom resolution. A case of invasive thymoma extending into the SVC and right atrium (RA) with SVC syndrome is reported. The patient underwent a combined resection of the invasive tumor and SVC under cardiopulmonary bypass (CPB), and the SVC and bilateral brachiocephalic vein (BCV) were reconstructed with an autologous pericardial ‘Y’ conduit. After 40 months of follow-up, the patient showed a patent graft and no tumor recurrence.

## Background

Although invasive thymoma commonly infiltrates neighbouring mediastinal structures, its extension into the superior vena cava (SVC) and consequent SVC occlusion are rare. In such cases, the urgent removal of the thymoma and radical resection of the infiltrated SVC representreasonable options, since induction therapy is time-consuming and useless for symptom resolution. A case of invasive thymoma extending into the SVC and right atrium (RA) with SVC syndrome is reported.

## Case presentation

**Figure 1:**
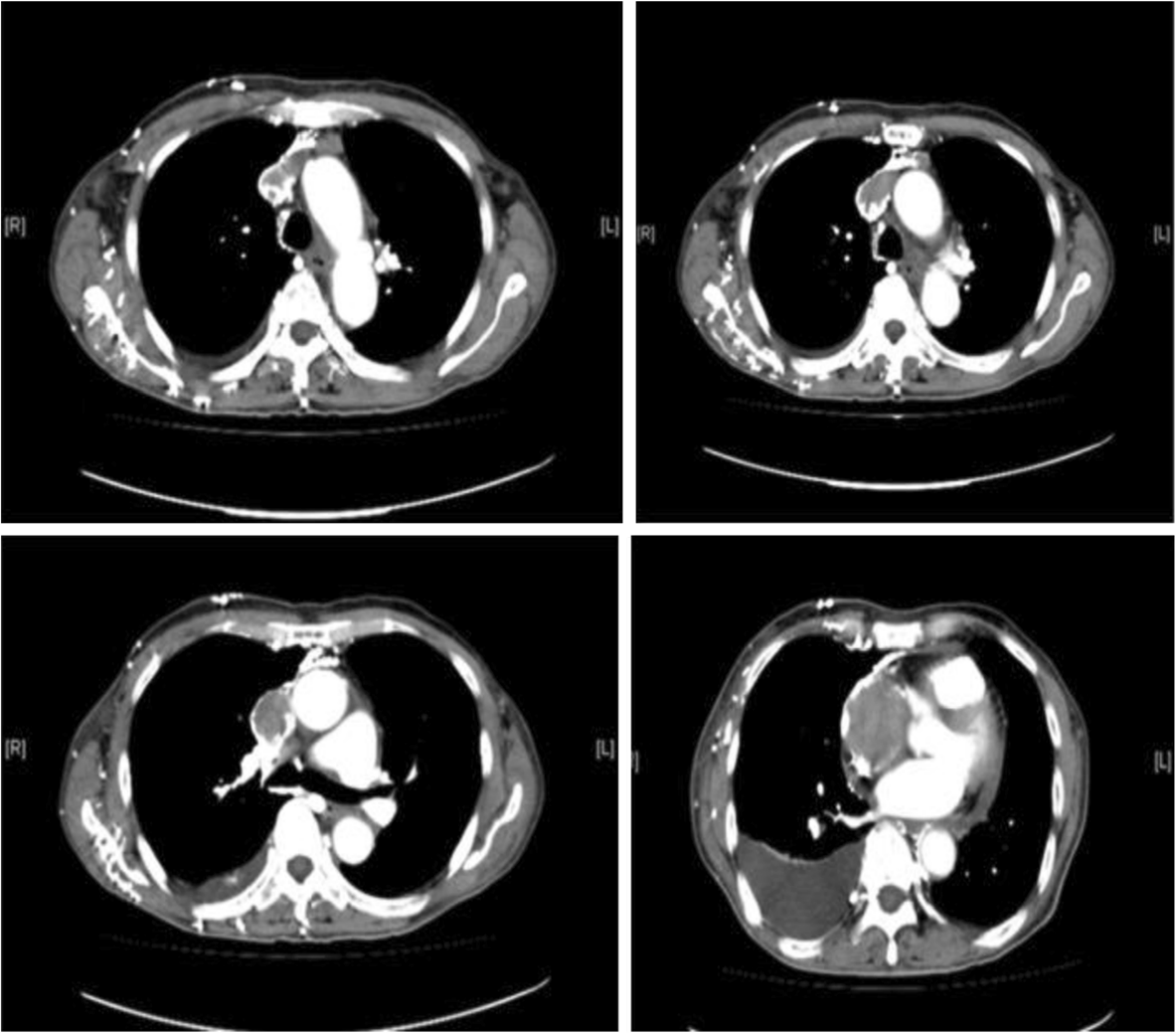
Computed tomographicscan revealed a large, well-defined, round softtissue density mass in the left brachiocephalic vein, superior vena cava, and right atrium.

A 75-year-old male patient presented to the Department of Cardio-Thoracic Surgery (Wuhan General Hospital of Guangzhou Military Command, Hubei, China)with signs and symptoms of SVC syndrome. During examination, an important jugular vein engorgement, edema of the face, neck, and anterior chest wall, and shortness of breath were found. A chest computed tomographic (CT) scan revealed a mass in the middle mediastinum, extending to the SVC and RA (Figure 1). Transthoracic echocardiography (TTE) examination demonstrated a hyperechogenic softtissue density mass in the RA, and no blood flow signal was found in the SVC or anonymous veins.

**Figure 2:**
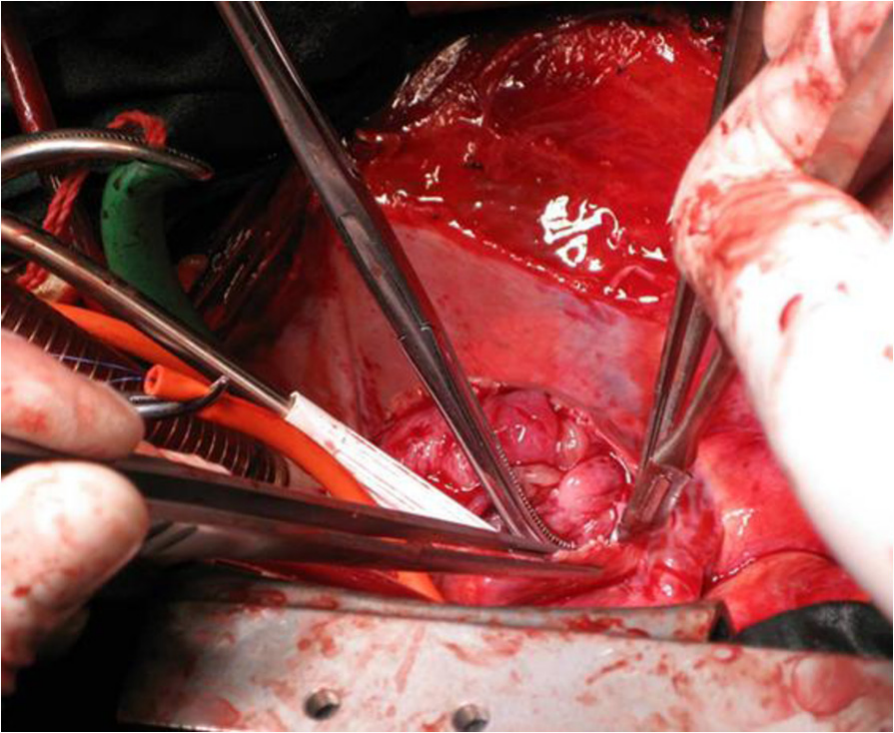
A large mass measuring 8 cm × 6 cm × 5 cm in the right atrium.

**Figure 3:**
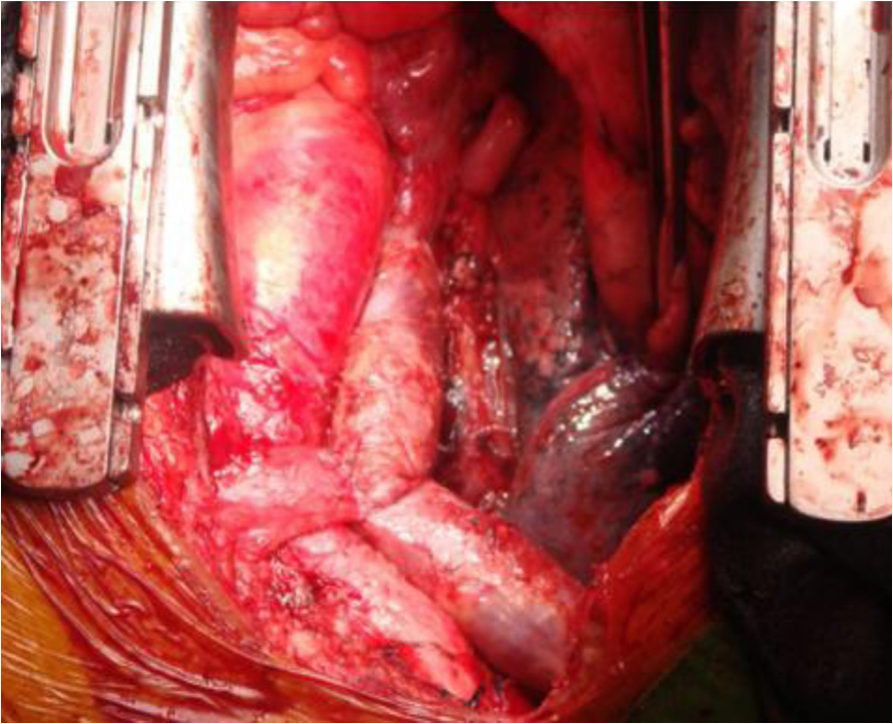
The superior vena cava and bilateral brachiocephalic vein were reconstructed with an autologous pericardial ‘Y’ conduit.

Due to its extensive nature and the potential risks associated with the location of the mass, the decision was made to take the patient to the operating room immediately without any preoperative chemotherapy or radiation therapy, to avoid neoplastic embolization and to resolve the SVC syndrome. The patient underwent median sternotomy, and it was observed that the thymoma had invaded the SVC and anonymous veins. A patch of pericardium was excised laterally to the left and right phrenic nerves. A radical thymectomy was performed. Cardiopulmonary bypass (CPB) was instituted after cannulation of the inferior vena cava (IVC) and the ascending aorta. The RA was then opened. A large mass (measuring 8 cm × 6 cm × 5 cm) was seen in the RA with its tail in the atrial septum (Figure 2). The intracardiac portion of the mass was resected. The SVC and brachiocephalic vein (BCV) were then opened. The tumor was firmly adherent to the vascular wall. The involved parts of the SVC and left BCV were removed. The pericardium was divided into a large square and a relatively smaller rectangle. The former was sutured to a 10 cm long conduit with 2 cm in diameter. The latter was sutured into a shorter conduit and anastomosed to the 10 cm conduit in an end-to-side way to complete the construction of the autologous pericardial ‘Y’ conduit. The bilateral BCV was then bypassed by anastomosing the autologous pericardial ‘Y’ conduit to the right atrial appendage. The left BCV was first cross-clamped and then the venousgraft anastomosis was made in an end-to-end way. The left BCV was then bypassed by anastomosing the autologous pericardial ‘Y’ conduit to the right atrial appendage. After the left-sided venous drainage had been reestablished, the right BCV was anastomosed to an autologous pericardial ‘Y’ conduit in the same way (Figure 3). All the anastomoses were made with continuous 5–0 prolenesutures (Ethicon, Inc, Somerville, NJ, USA). The patient was weaned off the CPB uneventfully.

**Figure 4:**
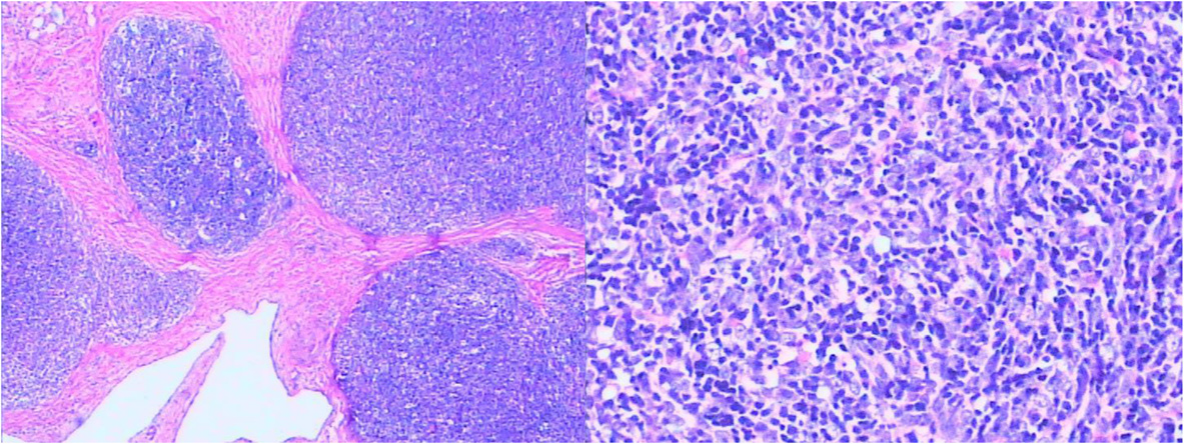
Pathologic examination revealed a type B3 thymoma.

Pathologic examination revealed a type B3 thymoma (Figure 4), which had directly invaded the left BCV, the SVC, and the RA. The patient was discharged home on postoperative day 14 without symptoms, and further management through outpatient chemotherapy was pursued. After the operation, the patient was anticoagulated with warfarin for 3 months, and then took aspirin instead of warfarin for anticoagulation. To date, 40 months after the surgical procedure, the patient is still alive and free of disease. In July 2013, a review of the chest CT showed that the autologous pericardial ‘Y’ conduit was patent, and the patient was free from any signs and symptoms indicative of SVC syndrome.

## Discussion

Invasive thymomas frequently invade neighbouring organs including the lung, heart, and large vessels, and in rare cases cause SVC syndrome. Invasive thymoma extending into the SVC and RA is rare. The results of surgical treatment for invasive thymoma extending into the SVC and RA have been reported by various institutions [1-5]. The goal of surgery in such cases is to restore the blood flow from the BCV to the RA and to completely resect the tumor. This can be performed either by cleaning the SVC and RA from the tumor bulk or through extra-anatomical grafts. Both biologic and synthetic materials, such as the autologous vein, autologousor bovine pericardium, and expanded polytetrafluoroethylene (PTFE) have been used in the venous system. However, the problem of flexion and kinking are prominent with long conduits. We used an autologous pericardial ‘Y’ conduit for reconstruction of the BCV and SVC. After 40 months of follow-up, the patient showed a patent graft. Although chemotherapy, radiation therapy, or both are effective, complete en bloc resection is indispensable for improving the outcome of patients. Combined venous and atrial wall resections are usually necessary because of the invasive growth pattern of this tumor. We report a rare case with invasive thymoma to the SVC and RA that underwent en bloc excision of the tumor with SVC being bypassed using an autologous pericardial ‘Y’ conduit with an excellent 40 months of patency. Use of an autologous pericardial ‘Y’ conduit in the venous system is most probably safe and at times may even be lifesaving. Reconstruction of the blood flow from the BCV to the RA, and complete en bloc resection obviously improve the outcome of these patients who suffer from invasive thymoma extending into the SVC and RA.

## Conclusions

Here, we report a rare case of a patient with invasive thymoma to the SVC and RA that underwent en bloc excision of the tumor with SVC being bypassed using an autologous pericardial ‘Y’ conduit with an excellent 40 months of patency. Use of an autologous pericardial ‘Y’ conduit in the venous system is most probably safe and at times may even be lifesaving. Reconstruction of the blood flow from the BCV to the RA and complete en bloc resection obviously improve the outcome of patients who suffer from invasive thymoma extending into the SVC and RA.

## Consent

Written informed consent was obtained from the patient for publication of this case report and any accompanying images. A copy of the written consent is available for review by the Editor-in-Chief of this journal.

## Abbreviations

BCV: Brachiocephalic vein; CPB: Cardiopulmonary bypass; CT: Computed tomographic; IVC: Inferior vena cava; RA: Right atrium; SVC: Superior vena cava; TTE: Transthoracic echocardiography.

## Competing interests

The authors declare that they have no competing interests.

## Authors’ contributions

YQD and JSL wrote the main manuscript, GLY, XMZ and JHX performed the operation, SBZ and TJ revised the manuscript for important intellectual content, and all authors gave final approval for the version to be submitted for publication. All authors read and approved the final manuscript.
